# Asymptotic entropy of the Gibbs state of complex networks

**DOI:** 10.1038/s41598-020-78626-2

**Published:** 2021-01-11

**Authors:** Adam Glos, Aleksandra Krawiec, Łukasz Pawela

**Affiliations:** 1 Institute of Theoretical and Applied Informatics, Polish Academy of Sciences, ul. Bałtycka 5, 44-100 Gliwice, Poland; 2 Institute of Informatics, Silesian University of Technology, ul. Akademicka 16, 44-100 Gliwice, Poland

**Keywords:** Quantum information, Computational science

## Abstract

In this work we study the entropy of the Gibbs state corresponding to a graph. The Gibbs state is obtained from the Laplacian, normalized Laplacian or adjacency matrices associated with a graph. We calculated the entropy of the Gibbs state for a few classes of graphs and studied their behavior with changing graph order and temperature. We illustrate our analytical results with numerical simulations for Erdős–Rényi, Watts–Strogatz, Barabási–Albert and Chung–Lu graph models and a few real-world graphs. Our results show that the behavior of Gibbs entropy as a function of the temperature differs for a choice of real networks when compared to the random Erdős–Rényi graphs.

## Introduction

A network represents a relationship among units of a complex system. The relations are encoded by edges while units are associated with nodes. Typical random graph models such as Erdős–Rényi graphs^1^ are usually not suitable for modeling real-world networks like the Internet^2^. Here complex network theory comes as a possible remedy. The boundary between a graph and a network is rather blurred, nevertheless a typical network is scale-free, small-world and has social structures. Typical examples of complex networks are Watts–Strogatz^3^ and Barabási–Albert networks^2^.

Graph entropy describes the graph in the context of evolution on it^4^. In classical walks one typically considers the von Neumann entropy calculated for the Laplacian, as Laplacian defines valid continuous-time stochastic evolution^5,6^. Studies on various types of graph entropy can be found in the literature^7^. The von Neumann entropy for complex networks was analyzed in^8,9^. Thermal state entanglement entropy on quantum graphs was studied in^10^. Entropy measure for complex networks using its Gibbs state was defined in^4^.

In contrary to stochastic evolution, continuous-time quantum walks accept arbitrary symmetric graph matrix which for undirected graphs includes adjacency matrix and normalized Laplacian^6,11,12^. Since it is known that the choice of a graph matrix does affect the evolution of quantum walk^11,12^, we claim that there is a need to design the entropy formula which accepts each of the above-mentioned matrices.

Entropy in the work^4^ is defined as the von Neumann entropy of Gibbs state of Laplacian matrix

1 $$\begin{aligned} S(\varrho _L^\tau ) = - {\mathrm {Tr}}\left( \frac{\exp (-\tau L)}{Z} \log \frac{\exp (-\tau L)}{Z} \right) , \end{aligned}$$

where *Z* is a normalizing constant. Formal introduction of this concept will be presented in the Preliminaries. Numerical calculations shed light on interesting behavior of the entropy depending on the parameter $\tau$ of the Gibbs state interpreted as a parameter proportional to the inverse of temperature^4,9^ or evolution time^4,9,13,14^. The authors of^4^ point the phase transition of entropy value for Erdős–Rényi and Watts–Strogatz graphs for some critical value $\tau _{\mathrm{crit}}$. Our analytical considerations on Erdős–Rényi graphs confirm that such a phase transition actually occurs, however, the value of $\tau _{\mathrm{crit}}$ depends on the graph order.

Depending on a graph, the phase transitions occurs either for smaller or larger values of $\tau$. The direction of phase transition change may be derived from the analysis of entropy limits of graphs with increasing graph order: when the entropy for fixed $\tau$ grows like $\log (n)$, then clearly the phase transition moves to the right. On the other hand, when entropy converges to zero, then the phase transition moves to the left.

For this reason, we calculated the entropy for some special graph classes for fixed parameter $\tau$ and changing graph order *n*. We made the entropy analysis for a few types of graph matrices, that is adjacency matrix, Laplacian and normalized Laplacian. It appeared that the entropy usually takes the form either *o*(1) or $\log n- O(1)$, which shows that the phase transition moves respectively to the left or right. Furthermore, the deviations from $\log n$ differ for different random graph models, which can give a clue about their properties. On top of that, we made a numerical analysis for the entropy of a few real-world graphs analyzing the location and the shape of its phase transition.

This work is organized as follows. We begin with preliminaries in “Preliminaries” section. Then, in “General entropy properties” section we present general theorems for entropy behavior basing on properties of the matrix spectra. The entropy values for specific graph classes are presented in “Entropy of specific graph classes” section. The entropy behavior studied for various random graph models and real-world graphs is described in “Random graphs” section. Eventually, conclusions can be found in “Conclusions” section.

## Preliminaries

We will be interested in studying the von Neumann entropy of Gibbs states associated with a graph *G*. A graph *G* is a pair (*V*, *E*) where *V* is a set of vertices and *E* is a set of edges. In this work we restrict ourselves to simple undirected graphs. A graph has three typical matrix representations: the adjacency matrix, the Laplacian matrix and the normalized Laplacian matrix. The adjacency matrix of a simple graph is a symmetric square matrix consisting of ones if two vertices are adjacent and zeros otherwise. The adjacency matrix of a graph *G* will be denoted *A*(*G*). The degree matrix is a diagonal matrix with degrees of vertices on the diagonal. The degree matrix will be denoted *D*(*G*). We will often make use of (combinatorial) Laplacian matrix which is defined as $L(G) :=D(G)-A(G)$. The normalized Laplacian is defined as  . When it will not make confusion we will be writing only ${\mathcal {L}}$ instead of ${\mathcal {L}}(G)$ and analogously for other graph matrices. Eigenvalues of matrices will be denoted $\lambda _{1} , \ldots , \lambda _n$, where $\lambda _{1} \ge \cdots \ge \lambda _n$.

In this paper we will use the big-O notation. Class *O*(*f*(*n*)) denotes a set of functions *g* such that there exist $c>0$ and $n_0\in {\mathbb {Z}}_{>0}$ s.t. for all $n\ge n_0$ we have $|g(n)|\le cf(n)$. We write $f(n)=\Theta (g(n))$ iff $f(n)=O(g(n))$ and $g(n)=O(f(n))$. Finally, class *o*(*f*(*n*)) denotes set of functions *g* s.t. $\lim _{n\rightarrow \infty } g(n)/f(n)=0$. In particular *O*(1) denotes a set of functions upperbounded in absolute value by a constant, and *o*(1) denotes a set of functions converging to 0.

Now we will introduce the von Neumann entropy of a quantum state $\varrho$. As $\varrho$ is a density matrix, it is positive and has unit trace, its eigenvalues form a probability vector. Thus, the von Neumann entropy of the state $\varrho$ is defined as the standard Shannon entropy of its eigenvalues. This fact can be succinctly written as

2 $$\begin{aligned} S(\varrho ) = -{\mathrm {Tr}}(\varrho \log (\varrho )) \end{aligned}$$

where $\log$ refers to the natural logarithm throughout this paper.

For any Hermitian operator *H* we can define an associated Gibbs state $\varrho _H^\tau$ as

3 $$\begin{aligned} \varrho _H^\tau = \frac{\exp (-\tau H)}{Z}, \end{aligned}$$

where $Z = {\mathrm {Tr}}(\exp (-\tau H))$ is the partition function^14^. The parameter $\tau$ can be regarded either as a parameter proportional to the inverse of the temperature^4,9^ or the diffusion time^4,9,13,14^. Note that the von Neumann entropy of the Gibbs state can be written as^4^

4 $$\begin{aligned} S(\varrho _H^\tau ) = \tau {\mathrm {Tr}}\left( H \varrho _H^\tau \right) + \log Z. \end{aligned}$$

This entropy has two simple properties summarized in the following lemma, which proof is stated in the Supplementary Materials in Section 1.

### **Lemma 1**

*Let* *H* *be a positive semidefinite matrix and* $c \in {\mathbb {R}}$. *It holds that* $S\left( \varrho _{cH}^\tau \right) = S\left( \varrho _H^{c\tau } \right)$ *and* $S\left( \varrho _{c{{1}}+ H}^\tau \right) = S\left( \varrho _H^{\tau } \right) .$

We will be writing $S\left( \varrho _H \right)$ instead of $S\left( \varrho _H^\tau \right)$ when the value $\tau$ does not need to be stated explicitly.

When calculating the entropy of a graph given by the adjacency matrix we will use the notation $S(\varrho _{A})$ for $S(\varrho _{-A})$. When dealing with the Laplacian and normalized Laplacian matrices we will be writing $S(\varrho _{L})$ and $S(\varrho _{\mathcal {L}})$ respectively.

Finally, let us present a simple proposition describing the limit behavior of graph entropy.

### **Proposition 2**

*Assume* *G* *is be a connected graph of order* *n*. *Then, for* $M\in \{A,L,{\mathcal {L}}\}$ *we have*

5 $$\begin{aligned} S(\varrho ^0_{M(G)}) = \log (n), \end{aligned}$$

6 $$\begin{aligned} \lim _{\tau \rightarrow + \infty } S(\varrho ^\tau _{M(G)}) = 0. \end{aligned}$$

The proof can be found in the Supplementary Materials in Section 2. In fact, the proof shows that even for not connected graphs the entropy converges to $\log (n)$ as $\tau \rightarrow 0$. On the other hand, for $\tau \rightarrow \infty$ for Laplacian and normalized Laplacian the entropy converges to $\log (k)$, where *k* is the number of connected components of *G*. For adjacency matrix the limit for non-connected graphs may depend on the form of connected components. Note that by the proposition for connected graph the entropy continuously changes from $\log (n)$ to zero, when $\tau$ changes from zero to infinity.

## General entropy properties

In this section we will present general theorems concerning the entropy’s behavior in which we assume only some restrictions on matrix spectra.

Let us begin with a proposition which shows a useful property of *d*-regular graphs. A *d*-regular graph is a graph whose all vertices have degree equal to *d*. For continuous-time quantum walk on *d*-regular graphs the evolution is independent on the choice of either adjacency matrix or Laplacian^6^. It follows from the fact that  and hence it affects only the global phase. For a similar reason, in the case of normalized Laplacian it can be seen as a change of time.

It turns out that the proposed entropy reflects this behavior.

### **Proposition 3**

*Let* *G* *be a* *d*-*regular graph. Then* $S(\varrho ^\tau _{A}) = S(\varrho ^\tau _{L})$ *and* $S(\varrho ^\tau _{\mathcal {L}}) = S(\varrho ^{\tau /d}_{A})$.

### *Proof*

Let *G* be a *d*-regular graph. Then Laplace matrix of *G* is  , where *A* is the adjacency matrix of *G*. Now from Lemma 1 we have that  .

The normalized Laplacian for the *d*-regular graph takes the form  . Therefore again from Lemma 1 we have
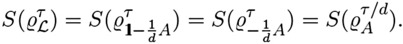
$$\square$$

It turns out that for the normalized Laplacian the entropy may take the values only from the very small interval. Let us first present a result for general Hermitian matrices with bounded spectra. Its proof can be found in the Supplementary Materials in Section 3.1.

### **Lemma 4**

*Let* *H* *be a matrix with eigenvalues bounded by* $c_1 \ge \lambda _i \ge c_2$. *Let* $\tau >0$ *be a constant. Then*

- *if* $$c_1,c_2\le 1/\tau$$, *then*
  8 $$\begin{aligned} \log n - S(\varrho _H) \le \tau (c_1 - c_2), \end{aligned}$$
- *if* $$c_2\le 1/\tau \le c_1$$, *then*
  9 $$\begin{aligned} \log n - S(\varrho _H) \le \tau \left( c_1- \min \{ c_1 \exp (\tau (c_2 - c_1)) , c_2 \} \right) , \end{aligned}$$
- *if* $$c_1,c_2\ge 1/\tau$$, *then*
  10 $$\begin{aligned} \log n - S(\varrho _H) \le \tau c_1 \left( 1- \exp \left( \tau (c_2 - c_1)\right) \right) . \end{aligned}$$

Conclusion directly drawn from the above Lemma is stated as a theorem concerning the entropy of a sequence of positive semidefinite matrices with finite spectral norm.

### **Theorem 5**

*Suppose* $(H_n)$ *is a sequence of positive semidefinite matrices* $n\times n$ *with spectral norm bounded by some constant independent* *of* *n*. *Then for fixed* $\tau$ *it holds that* $S(\varrho _{H_n}^\tau )=\log n- O(1)$.

For normalized Laplacian we have $c_2=0$ and $c_1=\Vert {\mathcal {L}}\Vert \le 2$^15^, which give us the situation as in Lemma 4. More specifically, independently on $\Vert {\mathcal {L}}\Vert$ and $\tau$ the bound yields

11 $$\begin{aligned} \log n - S(\varrho _{H}) \le \tau \Vert {\mathcal {L}}\Vert . \end{aligned}$$

The bound cannot be improved to $\log n-o(1)$ for general normalized Laplacians sequence of increasing size. In particular we will show that the deviation from $\log (n)$ occurs for a cycle, but also for all complex graphs considered in this paper, see “Random graphs” section.

Note that for Laplacian matrices of graphs with maximal degree $\Delta$ we have $\Delta \le \Vert L\Vert \le 2\Delta$^16^. Furthermore, for arbitrary graph we have $c_2=0$ for the Laplacian. Hence if a graph has a bounded degree, then we can simply utilize Theorem 5 in this scenario.

While considering Laplacian matrices we need to assume that a matrix is singular. More specifically, the number of zero eigenvalues is equal to the number of connected components of the graph. We will focus on the case when one of the eigenvalues is equal to zero and the rest of the eigenvalues are strictly positive (i.e. the graph is connected). In the next theorem we restrict ourselves to the case when all the nonzero eigenvalues converge to a positive constant.

### **Theorem 6**

*Let* *H* *be a singular nonnegative matrix of size* *n* *with single zero-eigenvalue and let* $\tau >0$ *be a constant. Assume that* $\lambda _1\rightarrow c$ *and* $\lambda _{n-1}\rightarrow c$ *for some constant* *c* *as* $n \rightarrow \infty$. *Then* $S(\varrho _{H})=\log n -o(1).$

The proof of the above theorem can be found in the Supplementary Materials in Section 3.2.

Now we focus on the case when the spectrum can be unbounded. An example of such a matrix is the Laplacian matrix. While it is singular and positive semidefinite, its norm coincides with the maximum degree of the graph, hence it can be unbounded. In the following theorem, proven in the Supplementary Materials in Section 3.3, we make an assumption only on the behavior of the smallest nonzero eigenvalue.

### **Theorem 7**

*Let* $H_n$ *be a singular nonnegative matrix of size* *n* *with single zero-eigenvalue and let* $\tau >0$ *be a constant. Assume* $\lambda _{n-1}(H_n) \gg \log n$. *Then* $S\left( \varrho _{H_n} \right) =o(1)$.

We use the notation $f(x) \gg g(x)$ when $\lim \nolimits _{x \rightarrow \infty }f(x)/ g(x) =\infty$.

The Laplacian matrix of a connected graph does not necessarily satisfy the assumption on $\lambda _{n-1}$ mentioned in Theorem 7, hence the result cannot be generalized into ‘arbitrary sequence of Laplacians’, even connected. As an example, the cycle graph $C_n$ of size *n* is known to have eigenvalues $2-2\cos (\frac{2\pi j}{n})$ for $j=0,\dots ,n-1$^17^. Hence the spectrum is bounded and we can apply Theorem 5. By this we have $S(\varrho _{L(C_n)}) = \log n -O(1)$. Such behavior shows the difference between Laplacian and normalized Laplacian in the sense of von Neumann entropy of the Gibbs state.

## Entropy of specific graph classes

In this section we study the entropy of a few selected classes of graphs. The entropy is calculated for three types of graph matrices: adjacency matrix *A*, Laplacian matrix *L* and normalized Laplacian ${\mathcal {L}}$. Four types of graphs were taken into consideration: empty graph, complete graph, bipartite graphs and cycle graph. An empty graph of order *n* is denoted by $E_n$. The symbol $K_n$ denotes the complete graph. A bipartite graph is a graph whose vertices are partitioned into two disjoint sets, *V* and *W*, and any two vertices from the same set cannot be adjacent. When a vertex $v \in V$ is adjacent to all vertices from the set *W* and vice-versa, then the graph is called a complete bipartite graph. Such a complete bipartite graph, where $|V|=n_1$ and $|W|=n_2$, is denoted by $K_{n_1,n_2}$. Finally, the symbol $C_n$ is used to denote a cycle graph.

**Table 1:** Asymptotic behavior of the entropy calculated for various graph classes described in “Entropy of specific graph classes” section.

	Adjacency matrix	Laplacian	Normalized Laplacian
$E_n$	$\log n-o(1)$	$\log n-o(1)$	–
$K_n$	*o*(1)	*o*(1)	$\log n-o(1)$
$K_{n_1,n_2}$	*o*(1)	Depends on $n_1,n_2$	
$K_{n_1,n_1}$	*o*(1)	*o*(1)	$\log n-o(1)$
$K_{n_1,1}$	*o*(1)	$\log n-o(1)$	$\log n-o(1)$
$C_n$	$\log n-\Theta (1)$	$\log n-\Theta (1)$	$\log n-\Theta (1)$

All the results are presented in Table 1. The proofs can be found in the Supplementary Materials in Section 4. An interesting observation is that in the first three cases the entropy behaves either like $\log n$ or converges to zero. For a cycle graph however the result is neither of them. More specifically, the entropy calculated for both adjacency and Laplacian matrices behaves in the same way

12 $$\begin{aligned} S(\varrho _{A(C_n)}) = S(\varrho _{L(C_n)})= \log n - 2 \tau \frac{I_1 (2\tau )}{I_0 (2 \tau )} +\log \left( I_0 (2\tau )\right) + o(1), \end{aligned}$$

where $I_\alpha (x)$ is the modified Bessel function of the first kind. For the normalized Laplacian of a cycle we obtain

13 $$\begin{aligned} S(\varrho _{{\mathcal {L}}(C_n)}) = \log n - \tau \frac{I_1 (\tau )}{I_0 ( \tau )} +\log \left( I_0 (\tau )\right) + o(1). \end{aligned}$$

It is also worth noting that the entropies calculated for adjacency matrix and Laplacian usually have the same asymptotic properties, that is either $\log n-o(1)$ or *o*(1). Nevertheless, we found an counterexample which is a star graph $K_{n_1,1}$ for which the entropy for adjacency matrix is substantially different than the entropy for Laplacian.

## Random graphs

In this section we consider various random graph models. Let us begin with Erdős–Rényi random graphs^1^. The symbol *G*(*n*, *p*) is used to denote a random graph of order *n* where the probability that any two vertices are adjacent equals *p*. A generalization of the Erdős–Rényi graph model is the Chung–Lu graph model^18,19^ in which we obtain a graph with a specified expected degree sequence $(w_1, \ldots , w_n)$. The probability that vertices $v_i$ and $v_j$ are adjacent equals $w_i w_j/\sum _k w_k$.

Watts–Strogatz random graphs^3^ are constructed as follows. In the first step we have a regular ring lattice, that is a graph of order *n* where each vertex is adjacent to *K* neighbors (*K*/2 on each side). Then, for each vertex we consider their neighbors from one side and rewire them with probability $\beta$ to some other vertex. Watts–Strogatz graphs are known to be small-world, meaning that in contrary to Erdős–Rényi graphs all vertices are close to each other. Nevertheless, the degree distribution is highly concentrated around *K*.

Barabási–Albert random graphs^2^ are constructed as follows. We begin with a complete graph with fixed order $m_0$. Then we add vertices one after another. Each time, a new vertex is adjacent to *m* of the already existing vertices. The probability that the new vertex is adjacent to the already-existing vertex *v* is proportional to the degree of the vertex *v*.

We will start with analytical results for Erdős–Rényi and Chung–Lu graphs for Laplacian and normalized Laplacian matrices. Then, we will present numerical results for other types of graph matrices and other graph models presented above.

### Erdős–Rényi graphs

The Laplacian matrix of a random Erdős–Rényi graph with $p \gg \log (n)/n$ almost surely has a single outlying zero eigenvalue and the rest of eigenvalues behaving like $np(1+o(1))$. A useful property of the second smallest eigenvalue is formulated as a theorem.

#### **Theorem 8**

^20^ *The second smallest eigenvalue* $\lambda _{n-1}$ *of the random Laplacian matrix* *L* *from Erdős-Rényi graph* *G*(*n*, *p*) *with* $p \gg \log (n)/n$ *satisfies a.a.s*.

14 $$\begin{aligned} \lambda _{n-1}=np +O(\sqrt{np\log n}). \end{aligned}$$

Moreover, from^12^ we have that $\lambda _1 \sim np$ for $p\gg \log (n)/n$. The next remark follows from Theorem 7.

#### *Remark 9*

The von Neumann entropy of Gibbs state of Laplacian of random Erdős–Rényi graph *G*(*n*, *p*) with $p \gg \log (n)/n$ converges a.a.s. to zero.

The main reason of such behavior is the strongly outlying 0 value. The behavior changes when $p=\Theta (\log (n)/n)$. For $p<(1-\varepsilon )\log (n)/n$ the graph is almost surely disconnected^1^, and since the dimensionality of the null-space of the Laplacian equals the number of connected components^17^, the graph entropy strongly depends on *n*.

Let us now consider the threshold behavior of Erdős–Rényi model when $p=p_0\frac{\log n}{n}$ with $p_0>1$. Here we have $\lambda _{n-1}\sim (1-p_0)W_{-1}^{-1}\left( \frac{1-p_0}{{\mathrm {e}}p_0}\right) \log n$^20^ and $\lambda _1\sim (1-p_0)W_{0}^{-1}\left( \frac{1-p_0}{{\mathrm {e}}p_0}\right) \log n$^12^, where $W_{-1},W_0$ are Lambert *W* functions. In this case the following theorem provides results for selected values of $\tau$. Its proof can be found in the Supplementary Materials in Section 3.4.

#### **Theorem 10**

*Let* $H_n$ *be a positive semidefinite matrix with a single* *zero-eigenvalue of size* *n* *and* $\tau >0$ *be a constant. Assume* $\lambda _{n-1}=a\log n$ *and* $\lambda _1=b\log n$ *for* $a,b>0$. *Then the behavior of the von Neumann entropy satisfies*

1. *if* $$\tau <\frac{1}{b}$$, *then* $$S(\varrho _{H_n})\ge (1-\tau b)\log n +o(1)$$,
2. *if* $$\tau =\frac{1}{b}$$, *then* $$S(\varrho _{H_n})\ge \log 2 +o(1)$$,
3. *if* $$\tau >\frac{1}{a}$$, *then* $$S(\varrho _{H_n})=o(1)$$.

For random Erdős–Rényi graphs the above theorem translates to the following remark.

#### *Remark 11*

Let $H_n$ be a Laplacian matrix of a random Erdős–Rényi graph for $p=p_0\frac{\log n}{n}$ with $p_0>1$. Then

1. if $\tau <W_{0}\left( \frac{1-p_0}{{\mathrm {e}}p_0} \right) /(1-p_0)$, then a.a.s. $S(\varrho _{H_n})\ge C\log n +o(1)$ for some $C \in (0,1)$.
2. if $$\tau > W_{-1}\left( \frac{1-p_0}{{\mathrm {e}}p_0} \right) /(1-p_0)$$, then a.a.s. $$S(\varrho _{H_n})=o(1)$$.

**Figure 1:**
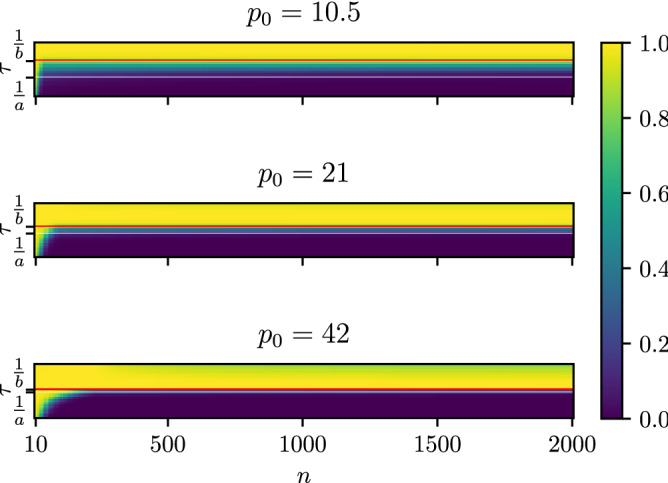
Entropy of the Gibbs state as a function of the parameter $\tau$ and the dimension of the graph, *n* for the Erdős–Rényi model. The value of the entropy is normalized by dividing by $\log n$. The phase transition can be easily seen. We show results for three values of the parameter $p_0$. The horizontal lines mark the theoretical boundaries for $\tau$ found in Theorem 10 and Remark 11. The red line marks $\tau =\frac{1}{b}$ while the white one corresponds to $\tau =\frac{1}{a}$.

**Figure 2:**
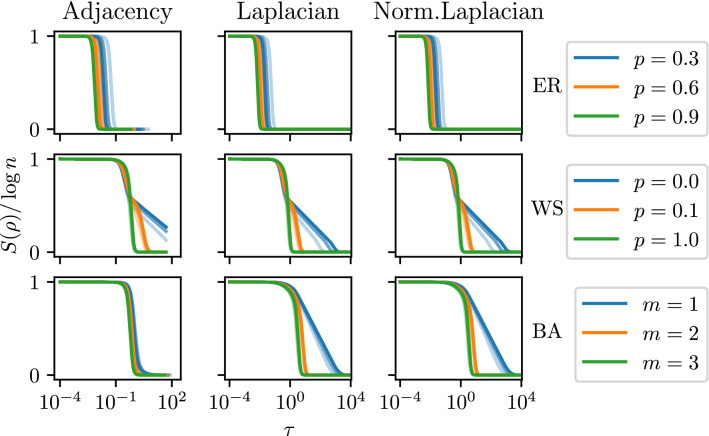
Illustration of the entropy’s phase transition for the ER, WS and BA models and all considered graph matrices. The value of the entropy is normalized by dividing by $\log n$. The dimension is $n=1200$. For WS we choose parameters $K=4$ and $\beta =0.6$. The specific model parameters are stated in the corresponding legends. Each plot is obtained by averaging 100 randomly sampled graphs. The transparency in the plot denotes the size of the subgraph of the originally sampled random graph. In increasing order of opacity, these lines correspond to 33%, 66% and 100% of the total number of nodes of the sampled graph chosen for calculations. Subgraphs were generated by choosing a vertex with the highest degree, and 33% and 66% vertices nearest to it.

Theorem 10 and Remarks 9, 11 give an analytical justification for the effect presented in^4^. The authors pointed that the phase-transition occurs with changing $\tau$. This phase transition is shown in Fig. 1, which shows the value of the entropy of the Gibbs state for an Erdős–Rényi graph with a function of the dimension of the graph and the parameter $\tau$. We show three values of the parameter $p_0$, namely $p_0=10.5, \; 21, \; 42$. To make it easier to compare the values for changing dimensionality, the value of the entropy is normalized by dividing by $\log n$. The phase transition is clearly visible. We should also note that for sufficiently large dimension *n* the normalized entropy does not depend on the dimension *n* around $\tau < \frac{1}{b}$. Yet, it still depends on $\tau$ as stated by Theorem 10. A more detailed view on this phenomenon is presented in Fig. 2. It depicts this phase transition for the ER, WS and BA models and for all considered graph matrices. The model specific parameters are stated in the legend.

Theorem 10 not only confirms that there is a strong correlation between spectral gap and the critical value of $\tau$ but also shows that the transition depends on the order of the graph *n*. Further numerical investigation shows that the entropy stabilizes with the graph order.

Let us now focus on the normalized Laplacian. It is known that normalized Laplacian of random Erdős–Rényi graph satisfies requirements of Theorem 6 for $p\gg \log (n)/n$^19^, however, we can go beyond that. The assumption can be relaxed to $pn=(1+\varepsilon )\log n$ for $\varepsilon >0$ by Corollary 1.2 from^20^. We conclude our results with the following remark.

#### *Remark 12*

Assume ${\mathcal {L}}$ is a normalized Laplacian matrix of random Erdős–Rényi graph with $p\ge (1+\varepsilon )\log n/n$. The von Neumann entropy of Gibbs state satisfies $S(\varrho _{{\mathcal {L}}}) - \log (n) \underset{{\text {a.a.s.}}}{\longrightarrow }0.$

### Chung–Lu graphs

By Theorem 4 from^19^, normalized Laplacian of a random Chung–Lu graph for which minimum expected degree $\omega _{\mathrm {min}} \gg \log n$ satisfies the requirement of Theorem 6. Therefore we have the following remark.

#### *Remark 13*

Assume ${\mathcal {L}}$ is a normalized Laplacian matrix of a Chung–Lu random graph for which minimum expected degree satisfies $\omega _{\mathrm {min}} \gg \log n$. The von Neumann entropy of Gibbs state satisfies $S(\varrho _{{\mathcal {L}}}) - \log (n) \underset{{\text {a.a.s.}}}{\longrightarrow }0.$

The following remark concerns the case of adjacency matrix of a Chung–Lu random graph. Its proof can be found in the Supplementary Materials in Section 3.5.

#### *Remark 14*

Let *A* be an adjacency matrix of a random Chung–Lu graph with the maximum expected degree satisfying $\omega _{\mathrm {max}}> \frac{8}{9}\log (\sqrt{2}n)$ and ${\tilde{d}} := \frac{\sum \omega _i^2}{\sum \omega _i} \gg \omega _{\mathrm {max}}\sqrt{\log n}$. Then $S(\varrho _A) = o(1)$.

### Numerical insight

In this section we will complement the analytical results from previous sections by numerical studies on various random graphs as well as some real-world graphs. Basing on the results in^4^ we expect that the information whether the graph describes real-world interactions may be distilled from the location and shape of the phase-transition.

We can clearly observe that the entropy function in $\tau$ differs among Erdős–Rényi graphs and Watts–Strogatz networks. Nevertheless, in the case of Erdős–Rényi and Barabási–Albert graphs we observe a similar shape of the plots with a single inflection point, however there is a difference in location. Furthermore, in Fig. 2 we also presented the shape of the curve for smaller graphs. We can see that for all values of *p*, the location of phase transition for Erdős–Rényi graphs goes to larger values of $\tau$, which is contrary to Watts–Strogatz and Barabási–Albert.

**Figure 3:**
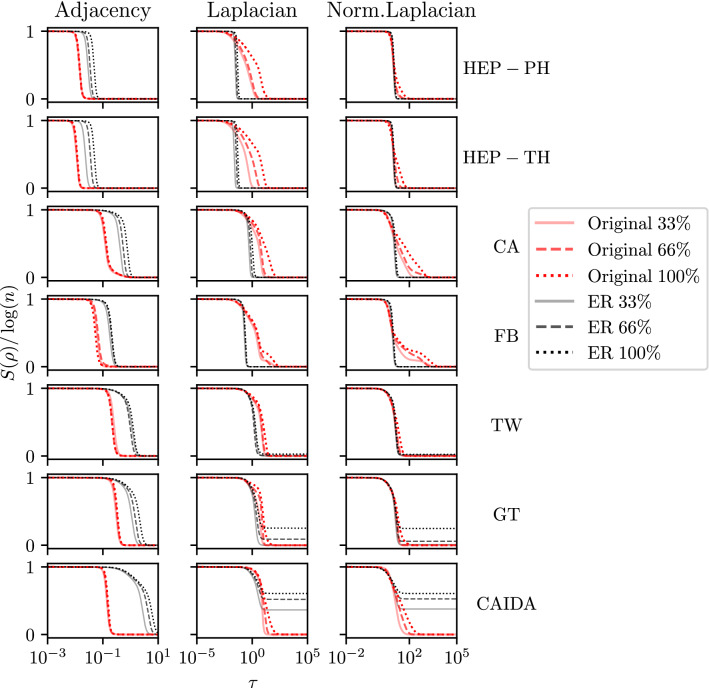
Illustration of the entropy’s phase transition for real-world graphs. Each graph was turned into a simple undirected graph by replacing directed edges with undirected edges. Then, for each graph we chose the largest connected component. For each real-world graph, we generated ten Erdős–Rényi graphs with parameter $p=2 m/n^2$ where *n*, *m* are the numbers of vertices and edges of the largest connected components of real-world graphs respectively. Finally, we took an average entropy. Subgraphs of real graphs were generated by choosing a vertex with the highest degree, and 33% and 66% vertices nearest to it.

We expect to observe similar situation for real-world graphs. More specifically, we focused on co-authorship graphs (HEP-PH, HEP-TH, CA)^21–23^, social networks (Facebook FB, Twitch TW)^24,25^, Gnutella graph (GT)^26^ and as-caida (CAIDA)^27^ graphs. All the plots are presented in the Fig. 3. Moreover, for the sake of comparison we considered Erdős–Rényi graphs chosen so that the number of vertices was the same as in the corresponding real-world graph and the expected number of edges equals the number of edges of the real-world graph. Finally, we also calculated the entropy of subgraphs of real graphs to analyze how the phase transition changes with the graph size.

For some graphs we observe nontrivial changes in the pace of entropy change, similarly to as it was in Watts–Strogatz graphs (see Fig. 2). This is the most prominent in the case of Facebook for Laplacian and normalized Laplacian, but for these matrices a similar effect can be observed also for HEP-PH, HEP-TH and GT. It is worth noting that these pace changes occur independently on the type of graph. More precisely, for co-authorship graphs the pace changes are clearly visible for HEP-PH and HEP-TH, while they are not visible for CA. This is even more appealing in the case of social network graph, that is the pace changes are very clear for FB graph while they are not visible for other graph.

For all real-world graphs for adjacency matrix, the phase transition occurs for larger values of $\tau$ than for the corresponding Erdős–Rényi graphs. Contrary to adjacency matrix, for Laplacians and normalized Laplacians the phase transition starts roughly at the same value of $\tau$ for both real-world and Erdős–Rényi graphs. On the other hand, phase transitions usually are more rapid for random graphs. Different behavior can be observed for Erdős–Rényi graphs corresponding to GT and CAIDA graphs. In those cases Erdős–Rényi graphs have many disconnected components and therefore the limit as $\tau \rightarrow \infty$ is no longer zero.

Finally, there is almost no change in the shape and location of the phase transition of the entropy for real graphs for adjacency matrix. In contrary, for corresponding Erdős–Rényi graphs we observe that with the increasing number of nodes the location of the phase transition moves to higher values of $\tau$. In the case of the Laplacian matrix, we observe that the location of phase transition remains the same for Erdős–Rényi graphs, while for real graphs it clearly goes to larger values of $\tau$. Similar behavior is observed for the normalized Laplacian, however for some real graphs (HEP-PH, HEP-TH) it is less evident compared to the Laplacian. The only case for which the values of entropy was similar to the corresponding Erdős–Rényi graph is the entropy of real graph TW for normalized Laplacian. Finally, the non-trivial shape of the phase transition observed for FB can be found also for subgraphs of FB, however for HEP-PH and HEP-TH it is observed only for the original graph.

All the code used to obtain the results presented here is available on GitHub at https://github.com/iitis/graph-entropy.

## Conclusions

This work is focused on studying the entropy of the Gibbs state for various graphs. We made the analysis for three types of graph matrices: adjacency matrix, Laplacian and normalized Laplacian for various graph classes. It turns out that the asymptotic properties of the same graph may differ depending on which graph matrix is taken into consideration. We proved a few general theorems which assume only some constraints on matrix spectra. Moreover, we studied several graph classes like complete graphs, bipartite graphs and cycle graphs, and derived the formulas for their entropy. It turned out that entropy usually takes the values either $\log n -o(1)$ or *o*(1), which implies the shift of the phase transition.

We considered also various random graph models and real-world graphs. We focused on the phase transition in $\tau$ of the entropy of Erdős–Rényi, Chung–Lu, Watts–Strogatz, Barabási–Albert random graphs with fixed graph order and some real-world graphs from various domains like co-authorship and social networks. Analysis of real graphs shows that we can indeed distill the information whether the graph represents some real-world interactions. This can be distilled from the position and, in some cases, the shape of the plot. The exact nature of this shift is dependent on the chosen graph matrix, however for adjacency matrix and Laplacian the difference were most evident.

## Supplementary information

Supplementary Information.
