# Supplementary material for: Asymptotic entropy of the Gibbs state of complex networks
Source: Sci Rep. 2021 Jan 11;11:311. doi: 10.1038/s41598-020-78626-2 (PMC7801599; doi:10.1038/s41598-020-78626-2)
Supplement: Supplementary file 1 — Supplementary Information. [file 41598_2020_78626_MOESM1_ESM.pdf]

ASYMPTOTIC ENTROPY OF THE GIBBS STATE OF COMPLEX NETWORKS – SUPLEMENTARY MATERIALS

ADAM GLOS[1,2,*], ALEKSANDRA KRAWIEC[1] AND ŁUKASZ PAWELA[1]

[1]*Institute of Theoretical and Applied Informatics, Polish Academy of Sciences, ul. Bałtycka 5, 44-100 Gliwice, Poland*

[2]*Institute of Informatics, Silesian University of Technology, ul. Akademicka 16, 44-100 Gliwice, Poland*

[*] aglos@iitis.pl

1. Proof of properties of von Neumann entropy of the Gibbs state

Here we state the proof of claims made in Lemma 1.

Proof.

$$\begin{aligned} S(\varrho^\tau_{cH}) &= -\mathrm{Tr}\left(\frac{\exp(-\tau(cH))}{\mathrm{Tr}\exp(-\tau(cH))}\log\left(\frac{\exp(-\tau(cH))}{\mathrm{Tr}\exp(-\tau(cH))}\right)\right) \\ &= -\mathrm{Tr}\left(\frac{\exp(-(\tau c)H)}{\mathrm{Tr}\exp(-(\tau c)H)}\log\left(\frac{\exp(-(\tau c)H)}{\mathrm{Tr}\exp(-(\tau c)H)}\right)\right) = S(\varrho^{c\tau}_H). \end{aligned} \tag{15}$$

(16)

$$\begin{aligned} S(\varrho^\tau_{d\mathbb{1}+H}) &= -\mathrm{Tr}\left(\frac{\exp(-\tau(d\mathbb{1}+H))}{\mathrm{Tr}\exp(-\tau(d\mathbb{1}+H))}\log\left(\frac{\exp(-\tau(d\mathbb{1}+H))}{\mathrm{Tr}\exp(-\tau(d\mathbb{1}+H))}\right)\right) \\ &= -\mathrm{Tr}\left(\frac{\exp(-\tau d)\exp(H)}{\exp(-\tau d)\mathrm{Tr}\exp(-\tau H)}\log\left(\frac{\exp(-\tau d)\exp(H)}{\exp(-\tau d)\mathrm{Tr}\exp(-\tau H)}\right)\right) \\ &= -\mathrm{Tr}\left(\frac{\exp(H)}{\mathrm{Tr}\exp(-\tau H)}\log\left(\frac{\exp(H)}{\mathrm{Tr}\exp(-\tau H)}\right)\right) = S(\varrho^\tau_H). \end{aligned}$$

□

2. Entropy limits for $\tau \to 0$ and $\tau \to \infty$.

Assume we have a Hermitian matrix M.

2.1. $\tau = 0$.

$$S(\varrho^0_M) = -\mathrm{Tr}\left(\frac{\mathbb{1}}{n}\log\frac{\mathbb{1}}{n}\right) = \log n. \tag{17}$$

2.2. $\tau \to \infty$. Assume that $\lambda_1 \geq \lambda_2 \geq \ldots \geq \lambda_{n-k} > 0$ and $\lambda_{n-k+1}, \ldots, \lambda_n = 0$ are eigenvalues of M. Defining $\exp_i \coloneqq \exp(-\tau\lambda_i)$ and $\exp_{i,j} \coloneqq \exp(-\tau(\lambda_i - \lambda_j))$ we have

$$
\begin{aligned}
S(\varrho_M^\tau) &= \tau \mathrm{Tr}(M\varrho_M^\tau) + \log Z \\
&= \frac{\tau \sum_{i=1}^n \lambda_i \exp(-\tau\lambda_i)}{\sum_{i=1}^n \exp(-\tau\lambda_i)} + \log\left(\sum_{i=1}^n \exp(-\tau\lambda_i)\right) \\
&= \frac{\tau\lambda_1 \exp_1}{\sum_{i=1}^n \exp_i} + \ldots + \frac{\tau\lambda_{n-k}\exp_{n-k}}{\sum_{i=1}^n \exp_i} + \log\left(\sum_{i=1}^n \exp_i\right) \qquad (18) \\
&= \frac{\tau\lambda_1}{\sum_{i=1}^n \exp_{i,1}} + \ldots + \frac{\tau\lambda_{n-k}}{\sum_{i=1}^n \exp_{i,n-k}} + \log\left(k + \sum_{i=1}^{n-k} \exp_i\right) \\
&\underset{\tau\to\infty}{\longrightarrow} \log k
\end{aligned}
$$

where the limit follows from observing that for the j-th factor the nominator grows like τ, while in the denominator $\exp_{n,j}$ exponentially tends to infinity. More specifically

$$
\lim_{\tau\to\infty} \exp_{i,j} = \begin{cases} 0, & i < j \\ 1, & i = j \\ \infty, & i > j. \end{cases} \qquad (19)
$$

Finally, it suffices to note that $\lim_{\tau\to\infty} \exp_i = 0$ for every $i > 0$.

Let us consider Laplacian and normalized Laplacian matrices. Since the number of eigenvalues equal to zero is equal to the number of connected components, then for $k = 1$ we obtain the limit $\log(1) = 0$. In the case of adjacency matrix, we can always shift the matrix by $\lambda_1(-A)\mathbb{1}$ without the change of the entropy, see Lemma 1. Furthermore, for connected graphs there is a nonzero gap between the largest and second largest eigenvalue of the adjacency matrix which, similarly as in previous case, gives us $k = 1$, and hence the limit is zero.

3. Proofs of general theorems

3.1. Proof of Lemma 4.

Proof. The entropy takes the form

$$
\begin{aligned}
S(\varrho_H) &= \tau \mathrm{Tr}(H\varrho_H^\tau) + \log Z \\
&= \frac{\tau \sum_{i=1}^n \lambda_i \exp(-\tau\lambda_i)}{\sum_{i=1}^n \exp(-\tau\lambda_i)} + \log\left(\sum_{i=1}^n \exp(-\tau\lambda_i)\right). \qquad (20)
\end{aligned}
$$

The numerator is a sum of eigenvalues mapped by $f(x) = \tau x \exp(-\tau x)$ function. The function takes its unique maximum at $x = 1/\tau$.

Let us begin with the case when $c_1, c_2 \leq \frac{1}{\tau}$. Then

$$
S(\varrho_H) \geq \frac{n\tau c_2 \exp(-\tau c_2)}{n\exp(-\tau c_2)} + \log(n\exp(-\tau c_1)) = \log n + \tau c_2 - \tau c_1, \qquad (21)
$$

and therefore

$$
\log n - S(\varrho_H) \leq \tau(c_1 - c_2). \qquad (22)
$$

If $c_1, c_2 \geq \frac{1}{\tau}$, then

$$\begin{aligned} S(\varrho_H) &\geq \frac{n\tau c_1 \exp(-\tau c_1)}{n \exp(-\tau c_2)} + \log\left(n \exp(-\tau c_1)\right) \\ &= \tau c_1 \left(\exp\left(-\tau c_1 + \tau c_2\right)\right) + \log n - \tau c_1 \\ &= \log n + \tau c_1 \left(\exp\left(\tau(c_2 - c_1)\right) - 1\right), \end{aligned} \tag{23}$$

and hence

$$\log n - S(\varrho_H) \leq \tau c_1 \left(1 - \exp\left(\tau(c_2 - c_1)\right)\right). \tag{24}$$

Assume finally that $c_2 \leq \frac{1}{\tau} \leq c_1$. In this case we have

$$\begin{aligned} S(\varrho_H) &\geq \frac{n\tau \min\{c_1 \exp(-\tau c_1), c_2 \exp(-\tau c_2)\}}{n \exp(-\tau c_2)} + \log\left(n \exp(-\tau c_1)\right) \\ &= \tau \min\left\{c_1 \frac{\exp(-\tau c_1)}{\exp(-\tau c_2)}, c_2 \frac{\exp(-\tau c_2)}{\exp(-\tau c_2)}\right\} + \log n - \tau c_1 \\ &= \log n + \tau\left(\min\{c_1 \exp(\tau(c_2 - c_1)), c_2\} - c_1\right), \end{aligned} \tag{25}$$

and therefore

$$\log n - S(\varrho_H) \leq \tau\left(c_1 - \min\{c_1 \exp(\tau(c_2 - c_1)), c_2\}\right). \tag{26}$$

□

3.2. **Proof of Theorem 6.**

Proof. The entropy takes the form

$$\begin{aligned} S(\varrho_H) &= \tau \mathrm{Tr}(H \varrho_H^\tau) + \log Z \\ &= \frac{\tau \sum_{i=1}^n \lambda_i \exp(-\tau \lambda_i)}{\sum_{i=1}^n \exp(-\tau \lambda_i)} + \log\left(\sum_{i=1}^n \exp(-\tau \lambda_i)\right). \end{aligned} \tag{27}$$

Since the matrix H is singular, we can extract a single zero eigenvalue. Hence the first part of the sum can be bounded as

$$\frac{\tau(n-1)\lambda_{n-1} \exp(-\tau \lambda_1)}{1 + (n-1)\exp(-\tau \lambda_{n-1})} \leq \tau \mathrm{Tr}(H \varrho_H^\tau) \leq \frac{\tau(n-1)\lambda_1 \exp(-\tau \lambda_{n-1})}{1 + (n-1)\exp(-\tau \lambda_1)} \tag{28}$$

Both bounds converge to τc and hence $\tau \mathrm{Tr}(H \varrho_H^\tau)$ as well converges to τc.

Similarly for $\log Z$ we have

$$\log(1 + (n-1)\exp(-\tau \lambda_1)) \leq \log Z \leq \log(1 + (n-1)\exp(-\tau \lambda_{n-1})) \tag{29}$$

or equivalently

$$\log\left(\frac{1}{n} + \frac{n-1}{n}\exp(-\tau \lambda_1)\right) \leq \log Z - \log n \leq \log\left(\frac{1}{n} + \frac{n-1}{n}\exp(-\tau \lambda_{n-1})\right) \tag{30}$$

which implies $\log Z - \log n \to -\tau c$ as $n \to \infty$, which finishes the proof. □

3.3. **Proof of Theorem 7.**

Proof. The entropy takes the form

$$\begin{aligned} S(\varrho_{H_n}) &= \tau \mathrm{Tr}(H_n \varrho_{H_n}^\tau) + \log Z \\ &= \frac{\tau \sum_{i=1}^n \lambda_i \exp(-\tau\lambda_i)}{\sum_{i=1}^n \exp(-\tau\lambda_i)} + \log\left(\sum_{i=1}^n \exp(-\tau\lambda_i)\right). \end{aligned} \tag{31}$$

Since H_n matrix is singular, we can extract a single zero eigenvalue.

First we consider $\tau\mathrm{Tr}(H_n\varrho_{H_n}^\tau)$. Since $x\exp(-x)$ is a decreasing function for $x > 1$ and since by assumption τ is constant and λ_{n-1} tends to infinity, we can bound

$$\begin{aligned} \tau\mathrm{Tr}(H_n\varrho_{H_n}^\tau) &\leq \frac{\tau(n-1)\lambda_{n-1}\exp(-\tau\lambda_{n-1})}{1+(n-1)\exp(-\tau\lambda_1)} \\ &\leq \tau(n-1)\lambda_{n-1}\exp(-\tau\lambda_{n-1}). \end{aligned} \tag{32}$$

Let $\lambda_{n-1} = \log(n)g(n)$, where $g(n) \gg 1$. Then

$$\tau(n-1)\lambda_{n-1}\exp(-\tau\lambda_{n-1}) = \tau(n-1)\log(n)g(n)n^{-\tau g(n)} \underset{n\to\infty}{\longrightarrow} 0. \tag{33}$$

Now we bound

$$\log Z \leq \sum_{i=1}^{n-1} \exp(-\tau\lambda_i) \leq (n-1)\exp(-\tau\lambda_{n-1}). \tag{34}$$

If $\lambda_{n-1} \gg \log n$, then the formula above tends to 0. Since both $\tau\mathrm{Tr}(H_n\varrho_{H_n}^\tau)$ and $\log Z$ converge to zero we have the result. □

3.4. **Proof of Theorem 10.**

Proof. The entropy takes the form

$$\begin{aligned} S(\varrho_{H_n}) &= \tau \mathrm{Tr}(H_n \varrho_{H_n}^\tau) + \log Z \\ &= \frac{\tau \sum_{i=1}^n \lambda_i \exp(-\tau\lambda_i)}{\sum_{i=1}^n \exp(-\tau\lambda_i)} + \log\left(\sum_{i=1}^n \exp(-\tau\lambda_i)\right). \end{aligned} \tag{35}$$

Since the matrix H_n is singular, we can extract single zero eigenvalue.

The $\log Z$ part can be bounded as

$$\begin{aligned} \log Z &\leq \log(1+(n-1)\exp(-\tau\lambda_{n-1})) \\ &= \log(1-n^{-\tau a}+n^{1-\tau a}), \end{aligned} \tag{36}$$

and

$$\begin{aligned} \log Z &\geq \log(1+(n-1)\exp(-\tau\lambda_1)) \\ &= \log(1-n^{-\tau b}+n^{1-\tau b}). \end{aligned} \tag{37}$$

Here behavior of $\log Z$ depends on τ parameter. If $\tau < \frac{1}{b}$, then $\log Z \geq (1-\tau b)\log n + o(1)$ and $\log Z \leq (1-\tau a)\log(n) + o(1)$. If $\tau > \frac{1}{a}$, then $\log Z$ converges to 0.

In the $\frac{1}{b} \leq \tau \leq \frac{1}{a}$ case we can provide partial results only. For $\tau = \frac{1}{b}$ we have $\log Z \geq \log 2 + o(1)$ and $\log Z \leq (1-\frac{a}{b})\log n + o(1)$. For $\tau = \frac{1}{a}$ we have $\log Z \leq \log 2 + o(1)$. For $\tau \in (\frac{1}{b}, \frac{1}{a})$ we can only provide $\log Z \leq (1-\tau a)\log n + o(1)$.

Since H_n is a nonnegative matrix, we have $\tau \mathrm{Tr}(H_n \varrho_{H_n}^\tau) \geq 0$. We can again provide simple bounds

$$\begin{aligned} \tau \mathrm{Tr}(H_n \varrho_{H_n}^\tau) &\leq \frac{\tau(n-1)\lambda_{n-1}\exp(-\tau\lambda_{n-1})}{1+(n-1)\exp(-\tau\lambda_1)} \\ &\leq \frac{\tau(n-1)a\log n \exp(-\tau a \log n)}{(n-1)\exp(-\tau b \log n)} \\ &= \tau a n^{\tau(b-a)} \log n, \end{aligned} \tag{38}$$

and similarly

$$\begin{aligned} \tau \mathrm{Tr}(H_n \varrho_{H_n}^\tau) &\geq \frac{\tau(n-1)\lambda_1 \exp(-\tau\lambda_1)}{1+(n-1)\exp(-\tau\lambda_{n-1})} \\ &\geq \frac{\tau(n-1)b\log n \exp(-\tau b \log n)}{n\exp(-\tau a \log n)} \\ &= \frac{n-1}{n}\tau b n^{\tau(a-b)} \log n. \end{aligned} \tag{39}$$

By combining the above inequalities we obtain the result. □

3.5. **Proof of Remark 14.**

Proof. Let $\lambda_n(-A) < 0$ be the single outlying eigenvalue of the matrix $-A$. By the use of Theorem 3 from [1] we have the bound

$$|\lambda_i(A)| \leq \sqrt{8\omega_{\max}\log(\sqrt{2}n)} \tag{40}$$

for $i = 1, \ldots, n-1$. From Lemma 1 we note that

$$S(\varrho_A) = S(\varrho_{-\lambda_n \mathbb{1}+A}) \tag{41}$$

and therefore it suffices to consider the case of a shifted spectrum with single zero eigenvalue and where for all the other eigenvalues we have

$$\lambda_i(-\lambda_n \mathbb{1} + A) = \lambda_i(A) + \lambda_n(-A) \geq \tilde{d} - 2\sqrt{8\omega_{\max}\log(\sqrt{2}n)}. \tag{42}$$

Using the assumption on $\tilde{d}$, asymptotically we obtain $\lambda_i(-\lambda_n \mathbb{1} + A) \gg \log n$ for $i = 1, \ldots, n-1$. Then we use Theorem 7. □

4. Entropy of specific graph classes - proofs

The analytical spectra of all the graph classes discussed in this appendix are taken from [2].

4.1. **Complete graph.**

The Laplacian matrix of the complete graph has a single eigenvalue equal to zero and $n-1$ eigenvalues equal to n. Therefore

(43)

$$\begin{aligned} S(\varrho_{L(K_n)}) &= \frac{\tau\sum_{i=1}^n \lambda_i \exp(-\tau\lambda_i)}{\sum_{i=1}^n \exp(-\tau\lambda_i)} + \log\left(\sum_{i=1}^n \exp(-\tau\lambda_i)\right) \\ &= n\tau\left(1 - \frac{1}{1+(n-1)\exp(-n\tau)}\right) + \log\left(1+(n-1)\exp(-n\tau)\right) \\ &= o(1). \end{aligned}$$

As the complete graph is a regular graph, then from Proposition 3 we have $S(\varrho_{L(K_n)}) = S(\varrho_{A(K_n)})$. In the case of normalized Laplacian we use the fact that the complete graph is a $(n-1)$-regular graph. Therefore the spectrum of the normalized Laplacian consists of $n-1$ eigenvalues equal to $\frac{n}{n-1}$ and a single eigenvalue equal to 0. Therefore we calculate

(44)
$$\begin{aligned} S(\varrho_{\mathcal{L}(K_n)}) &= \tau \frac{n \exp\left(-\tau \frac{n}{n-1}\right)}{1+(n-1)\exp\left(-\tau\frac{n}{n-1}\right)} + \log\left(1+(n-1)\exp\left(-\tau\frac{n}{n-1}\right)\right) \\ &= \log n - o(1). \end{aligned}$$

4.2. **Complete bipartite graph.** Now we study entropy of the complete bipartite graph Let us set $|V| = n_1$ and $|W| = n_2$. The spectrum of the adjacency matrix of such a complete bipartite graph K_{n_1,n_2} consists of $n_1 + n_2 - 2$ zero eigenvalues and $\pm\sqrt{n_1 n_2}$. Therefore we have

(45)
$$\begin{aligned} S\left(\varrho_{A(K_{n_1,n_2})}\right) &= \tau\sqrt{n_1 n_2}\left(1 - \frac{2\exp(\tau\sqrt{n_1 n_2}) + n_1 + m_2 - 2}{\exp(-\tau\sqrt{n_1 n_2}) + \exp(\tau\sqrt{n_1 n_2}) + n_1 + n_2 - 2}\right) \\ &\quad + \tau\sqrt{n_1 n_2} + \log\left(1 + \exp(-2\tau\sqrt{n_1 n_2}) + \frac{n_1+n_2-2}{\exp(\tau\sqrt{n_1 n_2})}\right) = o(1). \end{aligned}$$

The spectrum of Laplacian of the complete bipartite graph consists of a single 0 eigenvalue, $n_1 - 1$ eigenvalues equal n_2, $n_2 - 1$ eigenvalues equal n_1 and a single $n_1 + n_2$ eigenvalue. Now we assume $n_1 = n_2$ and calculate

$$\begin{aligned} S\left(\varrho_{L(K_{n_1,n_1})}\right) &= \tau n_1\left(1 - \frac{1-\exp(-2\tau n_1)}{1+2(n_1-1)\exp(-\tau n_1) + \exp(-2\tau n_1)}\right) \\ &\quad + \log\left(1 + 2(n_1-1)\exp(-\tau n_1) + \exp(-2\tau n_1)\right) = o(1). \end{aligned} \tag{46}$$

Assuming $n_2 = 1$ we obtain

$$\begin{aligned} S\left(\varrho_{L(K_{n_1,1})}\right) &= \tau\left(1 - \frac{1 - n_1\exp(-\tau(n_1+1))}{1+(n_1-1)\exp(-\tau)+\exp(-\tau(n_1+1))}\right) \\ &\quad + \log\left(1 + n_1\exp(-\tau) - \exp(-\tau) + \exp(-\tau(n_1+1))\right) \\ &\quad = \log(n_1+1) - o(1). \end{aligned} \tag{47}$$

Eigenvalues of a normalized Laplacian of a K_{n_1,n_1} graph consist of single eigenvalues equal 0 and 2, and $2n_1 - 2$ eigenvalues equal 1. Therefore

$$\begin{aligned} S\left(\varrho_{\mathcal{L}(K_{n_1,n_1})}\right) &= \tau\left(1 - \frac{1-\exp(-2\tau)}{1+(2n_1-2)\exp(-\tau)+\exp(-2\tau)}\right) \\ &\quad + \log\left(1 + (2n_1-2)\exp(-\tau) + \exp(-2\tau)\right) \\ &\quad = \log(2n_1) - o(1). \end{aligned} \tag{48}$$

Eigenvalues of a normalized Laplacian of a star graph $K_{n_1,1}$ consist of a single 0 eigenvalue, $n_1 - 1$ eigenvalues equal 1 and a single eigenvalue equal

2. Thus we have

$$
\begin{aligned}
S\left(\varrho_{\mathcal{L}(K_{n_1,1})}\right) &= \tau\left(1-\frac{1-\exp(-2\tau)}{1+\exp(-2\tau)+(n_1-1)\exp(-\tau)}\right)\\
&\quad+\log\left(1+\exp(-2\tau)+(n_1-1)\exp(-\tau)\right)\\
&= \log(n_1+1)-o(1).
\end{aligned} \tag{49}
$$

4.3. **Cycle graph.** Now we consider the cycle graph. We will prove Eq. (12) from the main part of the article. The eigenvalues of the adjacency matrix of the cycle C_n take the form $\lambda_j = 2\cos(\frac{2\pi j}{n})$ for $j = 0,\ldots,n-1$. Let $N_{\tau,n,j} := \exp\left(-2\tau\cos\left(\frac{2\pi j}{n}\right)\right)$. Then

(50)

$$
\begin{aligned}
S(\varrho_{A(C_n)}) &= 2\tau\frac{\sum_{j=0}^{n-1}\cos\left(\frac{2\pi j}{n}\right)N_{\tau,n,j}}{\sum_{j=0}^{n-1}N_{\tau,n,j}}+\log\left(\sum_{j=0}^{n-1}N_{\tau,n,j}\right)\\
&= 2\tau\frac{\frac{1}{n}\sum_{j=0}^{n-1}\cos\left(\frac{2\pi j}{n}\right)N_{\tau,n,j}}{\frac{1}{n}\sum_{j=0}^{n-1}N_{\tau,n,j}}+\log\left(n\frac{1}{n}\sum_{j=0}^{n-1}N_{\tau,n,j}\right)\\
&= 2\tau\frac{\frac{1}{n}\sum_{j=0}^{n-1}\cos\left(\frac{2\pi j}{n}\right)N_{\tau,n,j}}{\frac{1}{n}\sum_{j=0}^{n-1}N_{\tau,n,j}}+\log\left(\frac{1}{n}\sum_{j=0}^{n-1}N_{\tau,n,j}\right)+\log n.
\end{aligned}
$$

Now let us denote $x_j := \frac{j}{n}$. We calculate

$$
\begin{aligned}
\frac{1}{n}\sum_{j=0}^{n-1}N_{\tau,n,j} &= \sum_{j=0}^{n-1}\frac{1}{n}\exp\left(-2\tau\cos\left(2\pi x_j\right)\right)\\
&\underset{n\to\infty}{\longrightarrow}\int_0^1\exp\left(-2\tau\cos\left(2\pi x\right)\right)\mathrm{d}x = I_0(2\tau),
\end{aligned} \tag{51}
$$

where $I_\alpha(x)$ is the modified Bessel function of the first kind. Analogously we obtain

(52)

$$
\begin{aligned}
\frac{1}{n}\sum_{j=0}^{n-1}\cos(2\pi x_j)N_{\tau,n,j} &= \sum_{j=0}^{n-1}\frac{1}{n}\cos(2\pi x_j)\exp\left(-2\tau\cos\left(2\pi x_j\right)\right)\\
&\underset{n\to\infty}{\longrightarrow}\int_0^1\cos(2\pi x)\exp\left(-2\tau\cos\left(2\pi x\right)\right)\mathrm{d}x = -I_1(2\tau).
\end{aligned}
$$

Summing up, as

$$
\begin{aligned}
&2\tau\frac{\frac{1}{n}\sum_{j=0}^{n-1}\cos\left(\frac{2\pi j}{n}\right)N_{\tau,n,j}}{\frac{1}{n}\sum_{j=0}^{n-1}N_{\tau,n,j}}+\log\left(\frac{1}{n}\sum_{j=0}^{n-1}N_{\tau,n,j}\right)\\
&\underset{n\to\infty}{\longrightarrow}2\tau\frac{-I_1(2\tau)}{I_0(2\tau)}+\log\left(I_0(2\tau)\right),
\end{aligned} \tag{53}
$$

then for fixed τ we have

$$
S(\varrho_{A(C_n)}) = \log n - 2\tau\frac{I_1(2\tau)}{I_0(2\tau)}+\log\left(I_0(2\tau)\right)+o(1). \tag{54}
$$

As a cycle is a 2-regular graph, then from Proposition 3 we have that the same result will be obtained for the Laplacian matrix of a cycle.

To see why Eq. (13) from the main part of the article holds we note that as a cycle is a 2-regular graph, then $\mathcal{L}(C_n) = \frac{1}{2}L(C_n)$. Therefore it suffices to follow the proof of Eq. (12) from the main part of the article knowing that the eigenvalues of the normalized Laplacian are $\lambda_j = 1 - \cos(\frac{2\pi j}{n})$ for $j = 0, \ldots, n-1$.

References

[1] F. Chung and M. Radcliffe, "On the spectra of general random graphs," *The Electronic Journal of Combinatorics*, vol. 18, no. 1, p. 215, 2011.

[2] A. E. Brouwer and W. H. Haemers, *Spectra of graphs*. Springer Science & Business Media, 2011.
